# “Mimics” of Injuries from Child Abuse: Case Series and Review of the Literature

**DOI:** 10.3390/children11091103

**Published:** 2024-09-09

**Authors:** Martina Focardi, Valentina Gori, Marta Romanelli, Francesco Santori, Ilenia Bianchi, Regina Rensi, Beatrice Defraia, Rossella Grifoni, Barbara Gualco, Laura Nanni, Stefania Losi

**Affiliations:** 1 Forensic Medical Sciences, Department of Health Science, University of Florence, Largo Brambilla 3, 50134 Florence, Italy; martina.focardi@unifi.it (M.F.); valentina.gori@unifi.it (V.G.); marta.romanelli@unifi.it (M.R.); francesco.santori@unifi.it (F.S.); regina.rensi@unifi.it (R.R.); beatrice.defraia@unifi.it (B.D.); rossella.grifoni@unifi.it (R.G.); barbara.gualco@unifi.it (B.G.); 2 Laboratory of Personal Identification and Forensic Morphology, Department of Health Sciences, University of Florence, Largo Brambilla 3, 50134 Florence, Italy; 3 Pediatric Emergency Unit, Department of Intensive Care and Emergency, Meyer Children’s Hospital IRCCS, Viale Pieraccini 24, 50139 Florence, Italy; laura.nanni@meyer.it; 4 Responsible GAIA Service, Meyer Children’s Hospital IRCCS, Viale Pieraccini 24, 50139 Florence, Italy; stefania.losi@meyer.it

**Keywords:** child abuse, physical abuse, sexual abuse, child abuse mimics, mimickers, skin lesions

## Abstract

The phenomenon of child abuse/maltreatment is underestimated and often represents a difficult challenge for healthcare professionals and forensic pathologists who must proceed with the differential diagnosis with accidental or self-induced events, or with lesions due to pathologies that overlap with that of mistreatment, defined as “Mimics”. This study presents a case series with the aim of discussing lesions that may mimic signs of physical abuse in children but are due to a different etiology to raise awareness and train healthcare professionals and forensic pathologists on possible confounding factors in order to avoid diagnostic errors. Six cases of “Mimics” out of 418 cases of suspected mistreatment (1.43% of cases) were identified, presenting skin lesions initially classified as injuries of abuse due to their location and type and, in particular, sexual abuse for three cases. Then, the lesions and the subjects, in particular the anamnestic history, were examined by a multidisciplinary team and the diagnosis of *genital lichen sclerosus et atrophicus* in three cases, and the results of *popular healing techniques* (i.e., “cupping”) in the other three cases were ascertained. These situations require specific skills and a forensic background from healthcare professionals to conduct a correct differential diagnosis and the intervention of a multidisciplinary team to investigate every possible pathology or alternative therapeutic practice that could simulate child abuse. In particular, when “mimics” are due to alternative medicine, it should not strictly be considered child abuse, but professionals must be aware of the hypothesis of mistreatment in case of non-medical indication or potential personal injuries from other crimes, such as illegal practice of the medicine. This awareness is also crucial to direct the child toward appropriate medical care, and it is essential to recognize that these conditions can coexist within the same clinical presentation.

## 1. Introduction

As reported by the World Health Organization (WHO) and most recently by the literature, about one out of four children have suffered abuse or neglect in their life [1]. 

For health professionals, clinicians, and forensic pathologists, it is essential to recognize cases of abuse/mistreatment. This diagnosis is not always easy, given the differential diagnosis, which must be made with accidental, self-induced events, or harmfulness, which overlaps with mistreatment, i.e., the “Mimics”. 

According to the literature, in fact, there are clinical conditions and cultural practices, also defined as folk healing techniques, that are expressed in signs, mainly cutaneous, which, although harmless, can open up to many interpretative hypotheses [2]. 

This case series intends to present and discuss the lesions that may mimic signs of physical abuse in children and can be traced back to a different etiology, with the aim of raising awareness and training healthcare workers, clinicians and forensic pathologists, on possible confounding factors in order to avoid diagnostic errors.

## 2. Materials and Methods

The authors present 6 cases of suspected child victims of abuse/mistreatment, which came to the attention of the IRCCS Meyer’s health professionals. They were followed by the GAIA service (https://www.meyer.it/cura-e-sistenza/attivita-sanitarie/590-sportello-gaia, accessed on 20 July 2024), Childhood and Adolescent Abuse Group, consisting of a multidisciplinary team of specialists (pediatricians, forensic pathologists, psychologists, child neuropsychiatrists, nurses, social workers, etc.) that are dedicated to the protection of child’ rights and individuate cases of suspected child abuse/mistreatment.

The review of the literature was carried out using the Medline Database (PubMed; US National Library of Medicine—National Institute of Health) and free text protocols (e.g., “Mimics”, “Child Abuse”, “Mistreatment”, “Folk medicine”, “Cultural Practices”, “Cupping”, “Medical mimics”, “Lichen sclerosus et atrophicus”) individually combined through the Boolean operator “AND”. Filters such as full-text, publication date from 1988 to 2024, and English language were applied.

## 3. Results

**Figure 1:**
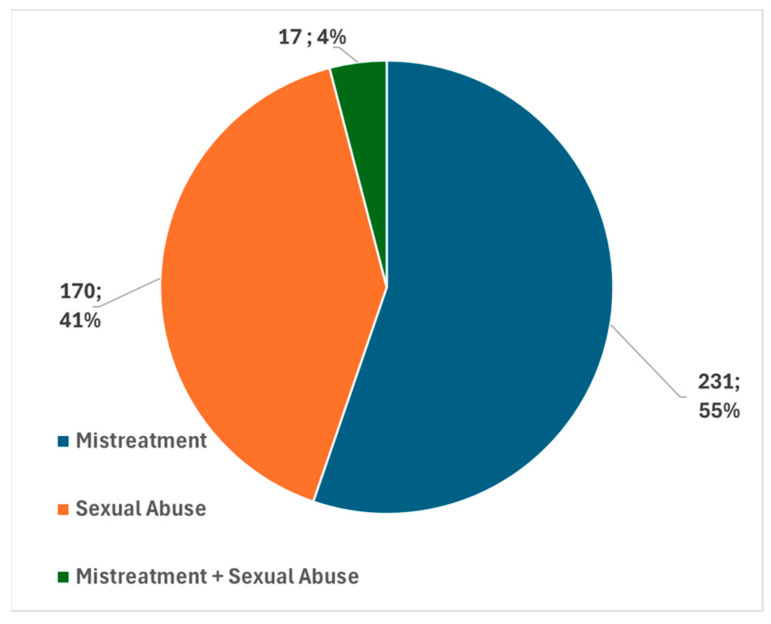
Percentages of suspected cases of mistreatment, sexual abuse, and mistreatment associated to sexual abuse reported at the GAIA service (Childhood and Adolescent Abuse Group) of the AOU Meyer IRCCS from 2015 up to 2022.

A total of 418 minors, aged from 0 to 18 years, suspected of maltreatment/sexual abuse observed at the GAIA service (Childhood and Adolescent Abuse Group) of the AOU Meyer IRCCS from 2015 to 2022 were analyzed (Figure 1).

Out of 418 cases, 6 cases of Mimics were identified (1.43% of cases). All the cases presented skin lesions, which, due to their location and type, were classified as suspicious of mistreatment; in three cases in particular, the suspicion was of sexual abuse (Table 1).

The cases were examined by a multidisciplinary team, allowing us to reach the diagnosis of genital *lichen sclerosus et atrophicus* in three cases, an idiopathic inflammatory pathology, which caused lesions and suspicious symptoms of sexual abuse. These lesions appeared as perigenital ecchymosis and genital abrasions with blood loss. In particular, a 4-year-old child was taken to IRCCS Meyer Children’s Hospital in Florence by teachers for the presence of suspected perigenital ecchymosis with bleeding.

The diagnostic process in these cases was expressed through an accurate history and physical examination conducted by experienced dermatologists and gynecologists.

Physical signs and physical examination are often suitable for a correct diagnosis for the experienced examiner, and histological evidence is not mandatory especially for children, as reported in the literature [3]. The biopsy is not usually performed in the anogenital area of children due to the possible physical and psychological invasiveness of the procedure often faced as a traumatic experience [3]. Furthermore, through a gynecological examination, the concomitant integrity of the hymen excluded the possibility of sexual abuse also being verified.

Three cases, however, were found to be attributable to two popular healing techniques: two were “cupping”, a common practice especially in oriental medicine and the other case concerned an alternative therapeutic practice commonly performed in Africa and performed by a “traditional healer” (Table 1). Such cases showed skin injuries such as burns/ecchymosis on the back or abrasions on the body with suspected distribution. In particular, an 8-month-old child was taken to IRCCS Meyer Children’s Hospital in Florence by her parents because she showed skin injuries as cigarette burns approximately 1 cm in size. The child had just returned from a trip to China with her grandparents, and upon her return, the parents were alarmed at the sight of these injuries.

The diagnostic process consisted of a circumstantial elements analysis with the help of a cultural mediator and a careful physical examination conducted by a multidisciplinary team.

## 4. Discussion

The increasing spread of child abuse makes it essential for clinicians and forensic clinical pathologists to intercept and correctly manage children suspected as victims of abuse through a specific multidisciplinary approach (physical, psychological, sexual, pathology of care).

In particular, one of the most common examples of child maltreatment is represented by physical abuse with cutaneous injuries as the most reliable proof of abuse.

Although the literature reports that cases of child abuse are often underestimated in that the damage caused by abuse is frequently misunderstood or incorrectly interpreted as accidental/self-inflicted [4], we cannot ignore those particular cases in which the lesions resemble and mimic injuries typical of abuse but which instead have another etiology, the so-called “Mimics”.

One multicentric prospective study investigated 2890 children suspected of physical abuse and found that mimics of physical abuse were detected in almost 5% of cases [5]. Additionally, skin injuries have been identified in almost half of the subjects with lesions mimicking physical abuse, approximately 2.4% [6,7].

Metz et al. [8] found that 51% of the mimics from a sample of 137 cases manifested with cutaneous injury, with or without an associated non-cutaneous component. The non-cutaneous mimics could include diseases involving multiple systems: metabolic bone disease (28%), hematologic/vascular (20%), infectious (16%), neurologic (9%), skeletal dysplasia (10%), oncologic (5%), gastrointestinal (2%), and other (10%) [7,8].

The correct recording and diagnosis of lesions have a fundamental role in the investigation of mistreatment or injuries in general, and it is important to intercept atypical and typical skin findings as accidental or resulting from abuse/mistreatment or attributable to other conditions.

Forensic activity has often shown that the injuries typically related to child abuse can have different causes, so it is important to thoroughly investigate and distinguish between findings that may result from abuse and the manifestations of benign injuries, congenital conditions, or acquired medical or popular therapeutic practices.

There are numerous alternative medical practices [9] that could leave cutaneous signs, such as bruising or burns, representing, although harmless, a diagnostic challenge, especially for examiners who do not come from countries where these techniques are usually practiced. In fact, these benign signs could “mimic” marks of physical abuse. Among these treatments, as reported in our cases, there is “cupping”, a practice that involves the sucking of some anatomical areas using special jars and leads to burns with a particular circular aspect similar to cigarette burns [2,9,10,11].

*Cupping* is a folk practice applied in many cultures to treat some illnesses, including pain, eating disorders, fever, and congestion [12]. The literature reports two different *cupping* methods. “Dry cupping” required a piece of cotton saturated in alcohol and lit in a cup to generate a vacuum. The cup is then applied to the skin creating ecchymotic lesions. Instead, “wet cupping” is performed on previously abraded skin with the same procedure; therefore, the resulting signs (rashes, petechiae, and burns) can be mistaken for cutaneous injuries of physical abuse [12].

Especially in cases in which the injury mimics signs of cigarette burns, it is important to exclude other possible causes to avoid a misdiagnosis of child abuse leading to an unjustified accusation with dramatic consequences. Another alternative therapeutic treatment that mimics cigarette burns is moxibustion [13], where fragments of the Artemisia plant, called “moxa”, are processed to form cones that are burned directly on the skin (direct moxibustion), or insulating materials are placed between the skin and the cone (indirect moxibustion) [14]. Many of these cases have multiple circular skin lesions distributed in a typical geometric pattern on the back [15]; therefore, it is crucial to distinguish them from cases of suspected maltreatment by deepening the anamnestic history of the child and performing a thorough, accurate objective examination.

With globalization and widespread immigration, health workers increasingly encounter patients from different customs. According to the literature, there are many other folk therapeutic practices that result in injuries that mimic the results of abuse [2,13]. One of these increasingly frequent practices is “Coining”, also known as “gua sha”. It is a Chinese practice that can cause worrisome signs when intercepted by inexperienced healthcare professionals [16]. This Asian therapeutic practice involves scraping of the skin, previously lubricated with warm massage oil, with a worn-down coin or soup spoon creating transient petechiae and ecchymosis on the skin [16], mimicking the sign of child abuse in the form of red streaks when applied on a child’s skin.

It should be considered that these alternative practices imported into countries other than those of origin are usually applied to adults rather than minors. It is therefore crucial that medical and forensic professionals are correctly informed of the incidence of these particular cases of mimics (50% of our reported cases), not only for the differential diagnosis of suspected abuse but also for the possible medico-legal implications that these practices applied to minors may have in the context of the legitimacy of the treatment.

In addition to the aforementioned practices, there are clinical conditions that mimic child abuse. Among these is, as shown above, Lichen sclerosus et atrophius, which is an exemplary imitator of sexual abuse.

*Lichen sclerosus et atrophicus* (LSAs) is a rare autoimmune inflammatory disease that shows genital lesions in most cases (about 95% of affected patients) and extragenital lesions in only 6–15% of cases [17,18]. The disease can occur in both sexes, but it mostly affects females, with a female-to-male ratio of 10:1. In those cases, the incidence shows a first peak during the prepubertal age and a second peak in postmenopause (usually diagnosed at 52–60 years old) [17,18].

The hymen is not usually involved in clinical conditions such as idiopathic LSA, unlike cases of sexual abuse in which physical signs often manifest in that anatomical position. Nevertheless, genital involvement and genital bruising or blistering should raise suspicion of sexual abuse and healthcare providers should be aware of the possible coexistence of LSA and sexual abuse.

Compared to cases of alternative treatments on minors, the literature is replete with medical mimics of child maltreatment [19], and there are several medical conditions that can be mistaken for child abuse. Among these, the most common are congenital and acquired skin conditions [6,17], metabolic bone disorders, and coagulation disorders [5].

As mentioned above, skin signs such as burns and bruises, represent “triggers” to suspect physical abuse. Congenital dermal melanocytosis, known as Mongolian spot or blue macule of infancy, is an absolutely benign condition with an irregularly shaped, bluish or greenish, non-painful skin lesion that appears in the neonatal period [20,21]. It is not infrequently mistaken for suspected child abuse since it is located primarily over the sacrum and buttocks, shoulders, arms, wrists, and ankles [20,21].

Less common, but still susceptible to mimicking child abuse, are vascular tumors, such as hemangiomas, and vascular malformations, which may manifest with skin lesions that are often circumscribed and well-defined (raised, red-brownish hyperplastic patches) but can sometimes ulcerate and be misinterpreted as child abuse [22,23].

Especially in the case of hemangiomas with ulcerative changes appearing on genitalia, a wrong diagnosis may lead a healthcare provider or caregiver to misinterpret the case and consider the child as a victim of abuse [22,23]. Therefore, a thorough history and detailed physical examination are necessary to rule out physical and sexual abuse of children.

However, there are numerous coagulation disorders, both congenital and acquired, which can cause damage that mimics physical abuse. These include hemophilia and von Willebrand’s disease [24], vitamin K deficiency, disorders of platelet function, including thrombocytopenia, hematological tumors such as leukemia [25], aplastic anemia, disseminated intravascular coagulation and several liver diseases [26].

Hereditary and acquired connective tissue disorders can also mimic child abuse. Among the hereditary ones, mention must be made of osteogenesis imperfecta (OI) [27], characterized by bone fragility that makes affected subjects more susceptible to fractures, and Ehlers–Danlos syndrome (EDS) [28,29], characterized by hypermobility of the joints and greater fragility of the skin and connective tissue.

Among the acquired forms, there is vitamin C deficiency, which causes fragility of the vessels and stroma, which presents with bruising and skin lesions.

Although the majority of children can be identified through clinical evaluation (sometimes supported by radiographic evaluation), a small percentage of children are misdiagnosed as abused without adequate laboratory testing [19], including, as demonstrated above, genetic analyses.

## 5. Conclusions

The phenomenon of “child abuse” is often underestimated, and injuries caused by abuse are often misunderstood or incorrectly interpreted as accidental/self-inflicted. On the other hand, we cannot ignore those particular cases in which the lesions resemble and mimic injuries typical of abuse, but which instead have another etiology. These situations require medical staff to have in-depth knowledge and a forensic background to consider alternative diagnoses that could simulate child abuse, as well as the intervention of a multidisciplinary team to investigate every aspect of the case. When “mimics” are connected to alternative therapeutic practices, although it is not child abuse in the strict sense, they must still be carefully considered, being able to evoke, in the case of a non-medical indication, the hypothesis of mistreatment [30], or also, depending on the type of practice, personal injuries from other crimes (e.g., the illegal practice of the medical profession).

A correct diagnosis is essential because interpretative errors can lead to serious consequences for children and their families.

Furthermore, quickly recognizing certain clinical conditions that are initially mistaken for child abuse is crucial to appropriate medical treatment, and it is essential that healthcare professionals are aware that these conditions can coexist within the same clinical presentation.

## Figures and Tables

**Figure 2:**
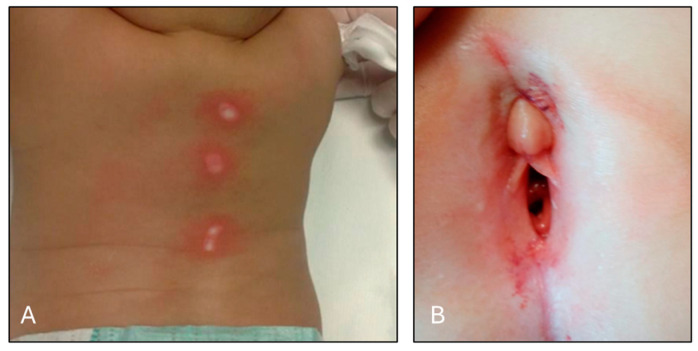
Panel (**A**): signs of burns on the back of a female child of 8 months due to cupping procedure; panel (**B**): perigenital ecchymosis with blood loss on a female child of 4 years due genital *lichen sclerosus et atrophicus*.

**Table 1:** Description of the 6 cases of “mimics”: the age, the type of lesions observed and the suspicion of abuse, and the differential diagnosis are reported.

Sex	Age	Country of Origin	Injuries Description	Suspected	Diagnosis
F	8 months	China	Burns on the back (Figure 2A)	Mistreatment	Cupping
M	3 years	Ghana	Abrasions and continuous solutions	Mistreatment	Alternative Therapeutic practice
M	6 years	Senegal	Ecchymosis on the back	Mistreatment	Cupping
F	4 years	Italy	Perigenital ecchymosis with blood loss (Figure 2B)	Sexual Abuse	Genital Lichen Sclerosus et Atrophicus
F	8 years	Italy	Perigenital ecchymosis and blisters	Sexual Abuse	Genital Lichen Sclerosus et Atrophicus
F	10 years	Albania	Genital abrasions with bleeding	Sexual Abuse	Genital Lichen Sclerosus et Atrophicus

## Data Availability

Not available.
